# Patients with low nicotinamide N-methyltransferase expression benefit significantly from bevacizumab treatment in ovarian cancer

**DOI:** 10.1186/s12885-021-07785-w

**Published:** 2021-01-14

**Authors:** Jun Li, Huiran Yue, Hailin Yu, Xin Lu, Xiaohong Xue

**Affiliations:** 1 Department of Gynecology, Obstetrics and Gynecology Hospital, Fudan University, No.419, Fangxie Road, Shanghai, 200011 China; 2 Shanghai Key Laboratory of Female Reproductive Endocrine Related Diseases, Shanghai, 200011 China

**Keywords:** Ovarian cancer, NNMT, Prognosis, Nomogram, Bevacizumab

## Abstract

**Background:**

The role of nicotinamide N-methyltransferase (NNMT) in ovarian cancer is still elusive. Our aim is to explore the expression of NNMT in ovarian cancer and to assess its association with patient prognosis and treatment response.

**Methods:**

We first analyzed the differential expression of NNMT among fallopian tube epithelium, primary ovarian cancers, metastatic ovarian cancers, and recurrent ovarian cancers using Gene Expression Ominus (GEO) database (GSE10971, GSE30587, GSE44104 and TCGA datasets). Then, we assessed the association of NNMT expression with clinical and molecular parameters using CSIOVDB database and GSE28739 dataset. Next, we evaluate the association of NNMT expression with the prognosis of ovarian cancer patients in both GSE9891 dataset and TCGA dataset. Finally, GSE140082 dataset was used to explore the association of NNMT expression with bevacizumab response.

**Results:**

NNMT expression was significantly elevated in lymphovascular space invasion (LVSI)-positive ovarian cancers compared with that in LVSI-negative ovarian cancers (TCGA dataset, *P* < 0.05), Moreover, increased expression of NNMT was associated with increased tumor stage, grade, and mesenchymal molecular subtype (CSIOVDB database). Survival analysis indicated that increased expression of NNMT was associated with a reduced OS in both GSE9891 dataset (HR: 2.28, 95%CI: 1.51–3.43, Log-rank *P* < 0.001) and TCGA dataset (HR: 1.55, 95%CI: 1.02–2.36, Log-rank *P* = 0.039). Multivariate analysis further confirmed the negative impact of NNMT expression on OS in ovarian cancer patients in those two datasets. Furthermore, the NNMT-related nomogram showed that NNMT shared a larger contribution to OS, compared with debulking status. More interestingly, bevacizumab conferred significant improvements in OS for patients with low NNMT expression (HR: 0.56, 95%CI: 0.31–0.99, Log-rank *P* = 0.049). In contrast, patients with high NNMT expression didn’t benefit from bevacizumab treatment significantly (HR: 0.85, 95%CI: 0.48–1.49, Log-rank *P* = 0.561). NNMT expression was positively correlated with the expression of genes, LDHA and PGAM1, involved in Warburg effect.

**Conclusions:**

In conclusion, NNMT expression is associated with the aggressive behavior of ovarian cancer, correlates with a poor prognosis, and is predictive of sensitivity to bevacizumab treatment.

## Background

Ovarian cancer, usually diagnosed at an advanced stage and characterized by metastatic bulky disease burden, has the highest mortality rate of all gynecologic cancer [1]. After debulking surgery and combined chemotherapy, most of the patients relapse and die from drug resistance. This underscores the significant clinical need to decipher the molecular biology of ovarian cancer and to identify new biomarkers to predict prognosis, which may be translated into personalized treatment strategies and survival improvements.

Nicotinamide N-methyltransferase (NNMT) is an S-adenosylmethionine-dependent enzyme, which plays a critical role in the biotransformation and detoxification of many drugs and xenobiotic compounds [2]. Overexpression of NNMT has been implicated in various cancers, including but not limited to colon, lung, hepatocellular, and bladder cancer [3–6]. In addition, increased expression of NNMT has been associated with tumor aggressiveness and demonstrated to facilitate the migration, invasion, viability, and proliferation of various cancer cells [3, 7–12]. Recently, NNMT has also been demonstrated to a master metabolic regulator of in several cancers and may be a therapeutically targeted [13, 14]. However, its role in ovarian cancer is still largely elusive [12, 13].

In present study, we analyzed the expression pattern of NNMT in ovarian cancer and evaluate its association with patient prognosis by taking advantage of the Gene Expression Omnibus (GEO) dataset and The Cancer Genome Altas (TCGA) dataset.

## Methods

### Database used in present study

The publicly available gene expression data used in our study is described in GEO database (GSE10971, GSE30587, GSE44104, GSE28739, GSE9891, and GSE140082). GSE10971 dataset was used to determine the differential expression of NNMT (probe ID:202237_at and 202238_s_at) between fallopian tube epithelium (FTE) (*n* = 24) and primary high grade serous tubal cancer/ovarian cancers (*n* = 13). GSE30587 dataset was used to determine the differential expression of NNMT (probe ID: 7943998) between paired primary ovarian cancer tissues(*n* = 9) and their omental metastasis counterparts(n = 9). GSE44104 dataset was used to determine the differential expression of NNMT (probe ID:202237_at and 202238_s_at) between ovarian cancer with recurrence (*n* = 20) and ovarian cancer with non-recurrence(*n* = 40). GSE28739 dataset was used to determine the differential expression of NNMT (probe ID: A_23_P127584) between drug sensitive ovarian cancer (*n* = 20) and drug resistant ovarian cancer (*n* = 30). If there are multiple probes all targeting NNMT, the average value of all probes is taken as an expression value of NNMT. Level 3 gene expression data with lymphovascular space invasion (LVSI) from TCGA dataset was used to explore the differential expression of NNMT between LVSI-positive ovarian cancers and LVSI-negative ovarian cancers.

CSIOVDB [15] (http://csibio.nus.edu.sg/CSIOVDB/CSIOVDB.html) was used to assess the association of NNMT expression with the clinical and molecular parameters of ovarian cancer.

GSE9891 dataset and TCGA dataset were used to develop and validated a NNMT-related nomograms to predict overall survival (OS) respectively. We only included patients with enough information (including age at initial diagnosis, histological grade, FIGO stage, debulking status, days to death, and survival status) in our final analysis. Totally, 242 patients in GSE9891 dataset and 462 patients in TCGA dataset were included. The expression value of NNMT expression together with the clinical information in GSE9891 dataset and TCGA dataset were extracted from the “curatedOvarianData” Bioconductor package (version 2.12 for R 3.0.3).

Kaplan-Meier analysis of OS for combination therapy of standard treatment and bevacizumab vs. standard treatment alone in ovarian cancer patients stratified by NNMT expression was performed in GSE140082 dataset.

### Statistical analysis

Detailed methods of statistical analysis were described in our previous published paper [16]. The expression of genes in GSE9891 and TCGA dataset were extracted from the curatedOvarianData R package. Since the present study was a single gene-centric study, we retained both the *P* value generated from student-t test and the adjusted *P* value generated from Limma R package for comparing gene expression between groups [17]. When using limma R package, the Benjamini-Hochberg multiple comparison adjustment was applied and the adjusted *P* value < 0.05 was considered statistically significant. “Low” and “High” were classified according to the gene expression level. The optimal cutoff values for NNMT, lactate dehydrogenase A (LDHA), and phosphoglycerate mutase 1 (PGAM1) expression were determined by the maximally selected rank statistics using R package survminer (http://www.sthda.com/english/rpkgs/survminer/). Correlation analysis was performed by Pearson method. All the statistical analyses were performed using IBM SPSS Statistics and R. *P* values < 0.05 are considered statistically significant.

## Results

### Differential expression of NNMT in fallopian tube epithelium (FTE), primary ovarian cancers (POCs)/primary tubal cancers (PTCs), metastatic ovarian cancers (MOCs), and recurrent ovarian cancers (ROCs)

**Fig. 1:**
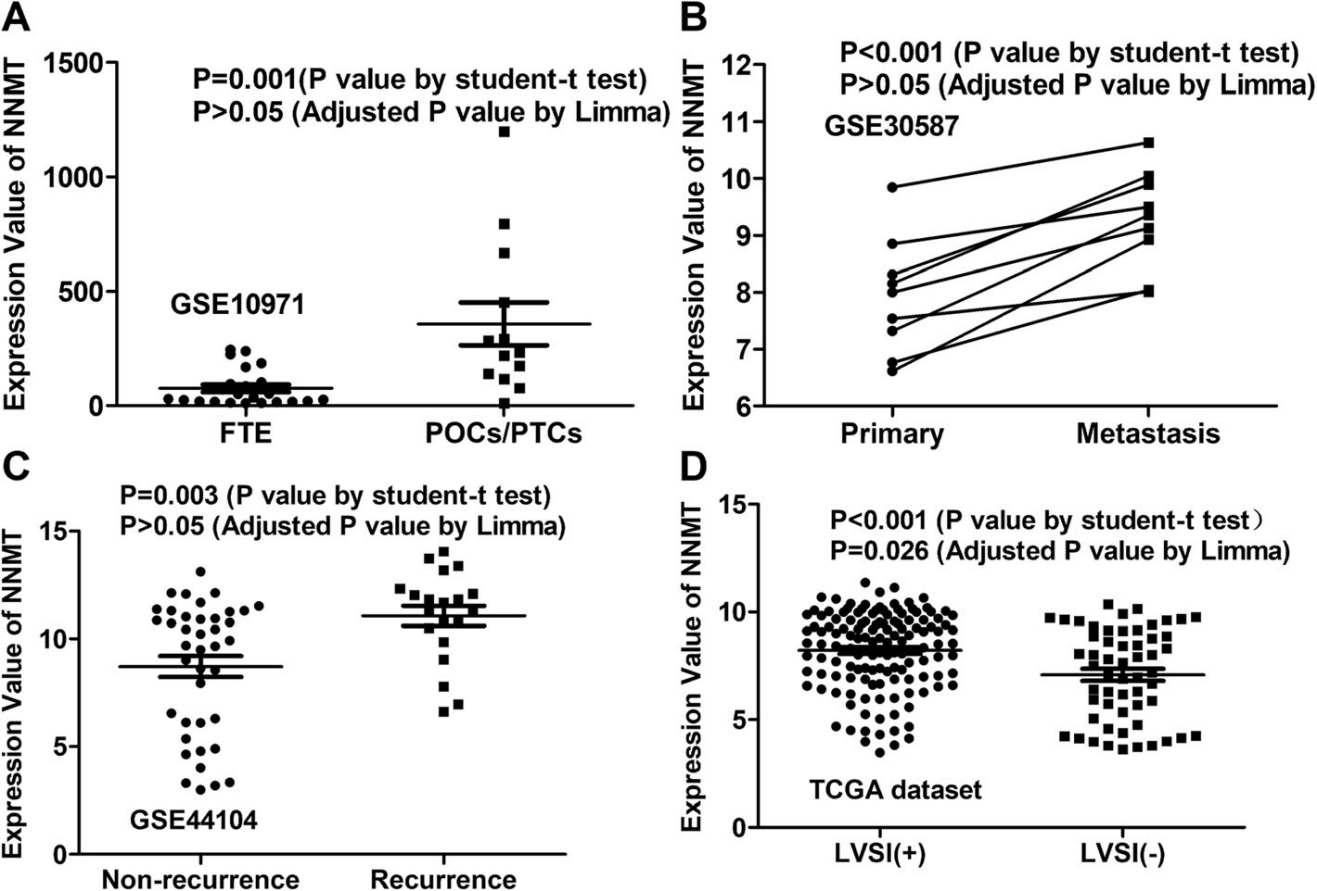
Expression pattern of NNMT in ovarian cancer tissues. **a** Differential expression of NNMT between FTs and POCs/PTCs. **b** Differential expression of NNMT between primary ovarian tumors and matched omental metastases. **c** Differential expression of NNMT between tumors with recurrence and tumors with no-recurrence. **d** Differential expression of NNMT between LVSI-positive ovarian cancers and LVSI-negative ovarian cancers

The mean expression value of NNMT was elevated in POCs/PTCs compared with that in FTs (Fig. 1a, GSE10971), and was increased in MOCs and ROCs compared with POCs (Fig. 1b, GSE30587; Fig. 1c, GSE44104). But the differences were not statistically significant suggested by adjusted *P* value generated from Limma R package. However, NNMT expression was significantly elevated in LVSI-positive ovarian cancers compared with LVSI-negative ovarian cancers (Fig. 1d, TCGA dataset, *P* < 0.05).

### The association of NNMT expression with clinical and molecular parameters

**Fig. 2:**
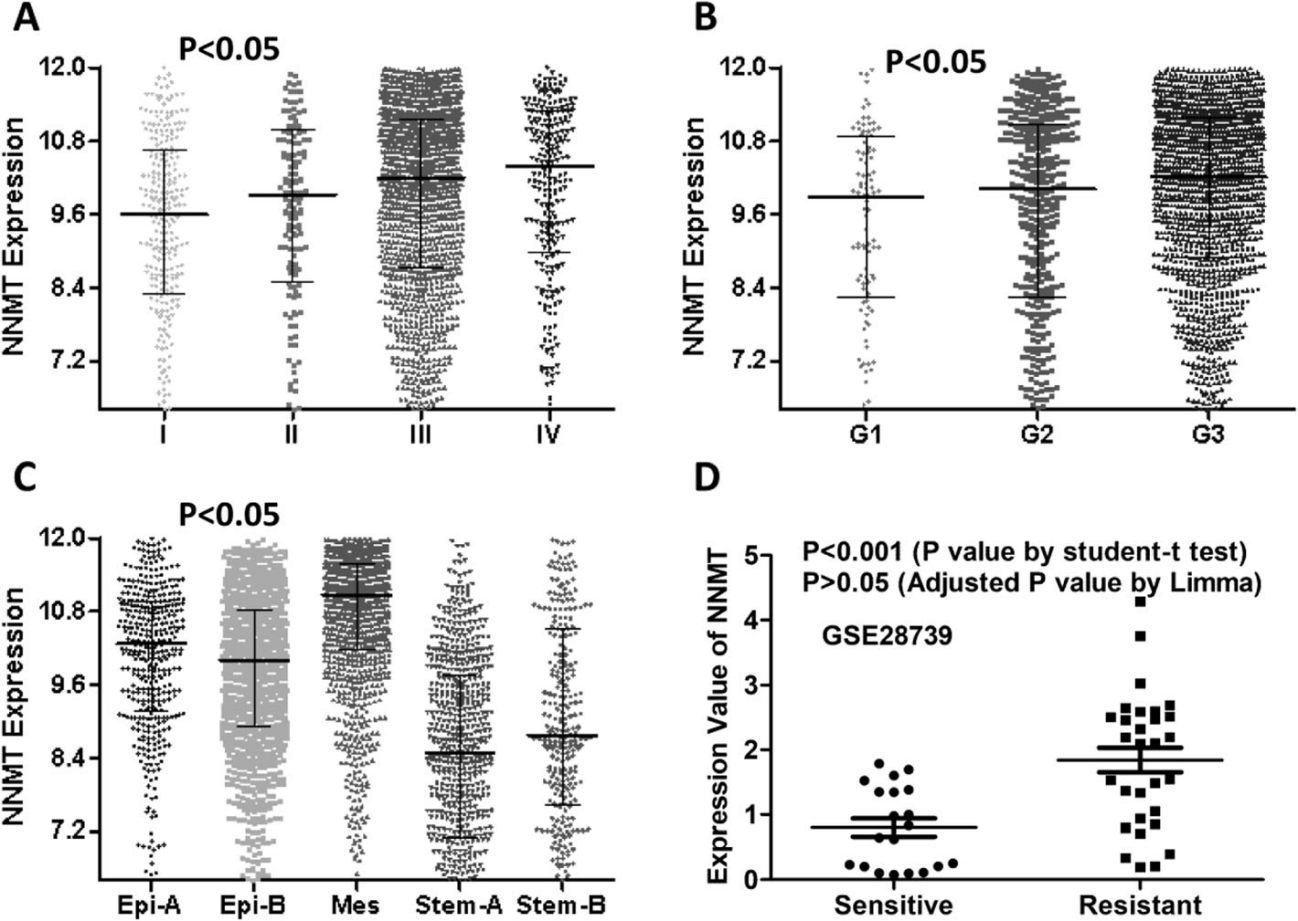
The association of NNMT expression with clinical and molecular parameters. **a**, **b** and **c** Increased expression of NNMT was associated with increased tumor stage (**a**), grade (**b**) and mesenchymal molecular subtype (**c**). **d** Differential expression of NNMT between drug-resistant and drug-sensitive ovarian cancers

Next, we try to explore whether NNMT expression was correlated with clinical and molecular parameters using CSIOVDB database. We showed that increased expression of NNMT was associated with increased tumor stage (Fig. 2a), grade (Fig. 2b), and mesenchymal molecular subtype (Fig. 2c). The mean expression value of NNMT was elevated in drug-resistant ovarian cancers compared with drug-sensitive ovarian cancers (Fig. 2d), but the difference was not statistically significant suggested by adjusted *P* value generated from Limma R package.

### The association of NNMT expression with OS

**Fig. 3:**
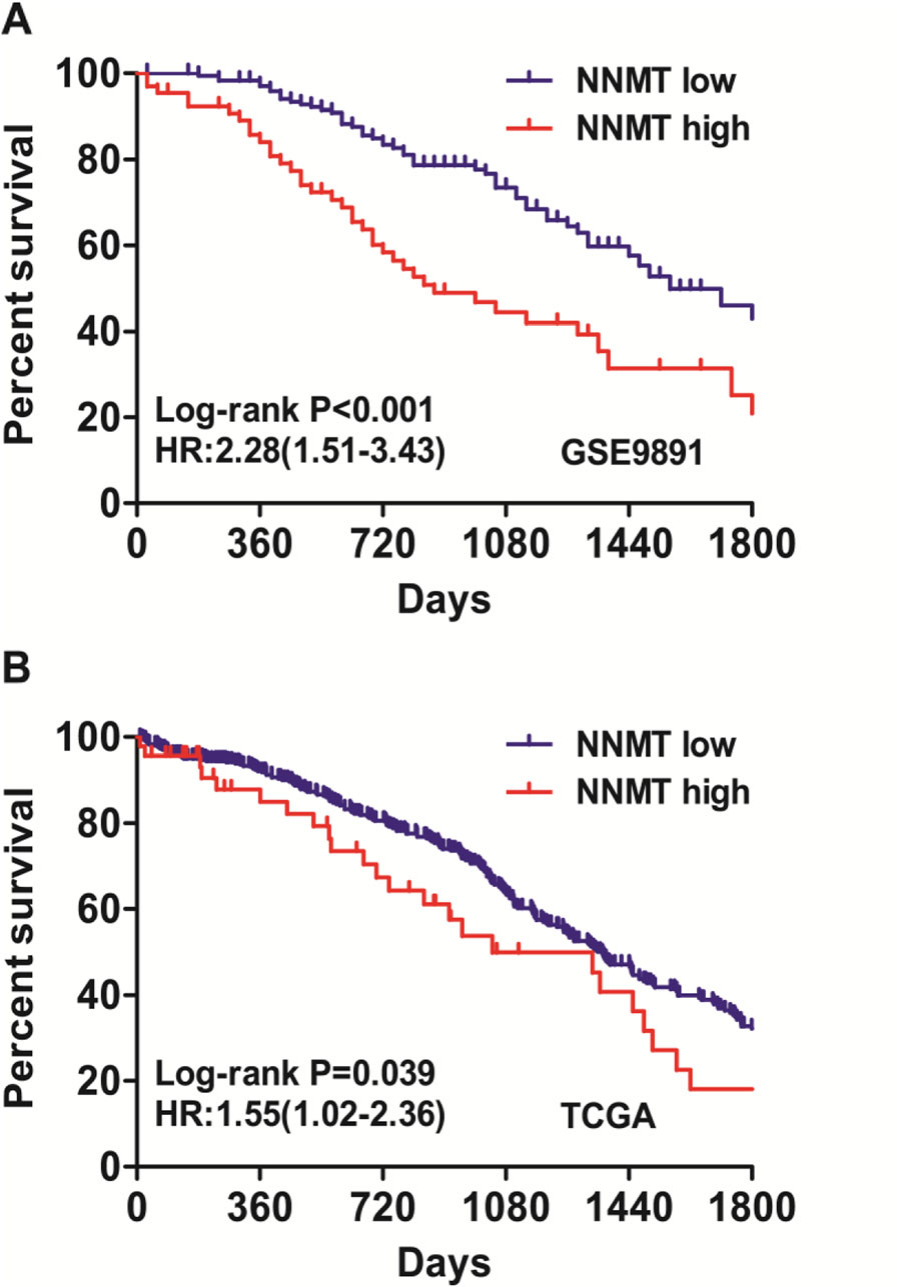
The association of NNMT expression with OS. **a** NNMT was associated with a shorter OS (HR: 2.28, 95%CI: 1.51–3.43, Log-rank *P* < 0.001) in GSE9891 dataset; **b** NNMT was associated with a reduced OS (HR: 1.55, 95%CI: 1.02–2.36, Log-rank *P* = 0.039) in TCGA dataset

Next, we evaluate the association of NNMT expression with the prognosis of ovarian cancer patients. Interestingly, survival analysis indicated that increased expression of NNMT was associated with a reduced OS in both GSE9891 dataset (Fig. 3a, HR: 2.28, 95%CI: 1.51–3.43, Log-rank *P* < 0.001) and TCGA dataset (Fig. 3b, HR: 1.55, 95%CI: 1.02–2.36, Log-rank *P* = 0.039). Multivariate analysis further confirmed the negative impact of NNMT expression on OS in ovarian cancer patients in both GSE9891 dataset (Table 1) and TCGA dataset (Table 2).

**Table 1:** The associations of NNMT expression with OS in GSE9891 dataset

Variables	Number of patients	Univariate analysis		Multivariate analysis	
		HR (95%CI)	*P* value	HR (95%CI)	*P* value
Age	242	1.029 (1.008–1.051)	0.006	1.031 (1.009–1.053)	0.005
Stage			0.002		0.012
Early	37	1		1	
Late	205	6.315 (1.997–19.963)		4.444 (1.379–14.322)	
Grade			0.142		/
Low	99	1		/	
High	143	1.365 (0.901–2.068)		/	
Debulking			0.005		0.054
Optimal	158	1		1	
Suboptimal	84	1.770 (1.190–2.633)		1.494 (0.993–2.249)	
NNMT expression			< 0.001		0.002
Low	176	1		1	
High	66	2.278 (1.513–3.430)		1.914 (1.265–2.894)

**Table 2:** The associations of NNMT expression with OS in TCGA dataset

Variables	Number of patients	Univariate analysis		Multivariate analysis	
		HR (95%CI)	*P* value	HR (95%CI)	*P* value
Age	462	1.024 (1.013–1.036)	< 0.001	1.022 (1.010–1.034)	< 0.001
Stage			0.001		0.004
Early	33	1		1	
Late	429	3.478 (1.639–7.381)		3.044 (1.415–6.550)	
Grade			0.048		0.269
Low	57	1		1	
High	405	1.438 (1.003–2.063)		1.230 (0.852–1.774)	
Debulking			0.030		0.304
Optimal	344	1		1	
Suboptimal	118	1.353 (1.030–1.776)		1.157 (0.876–1.528)	
NNMT expression			0.039		0.051
Low	415	1		1	
High	47	1.552 (1.022–2.357)		1.517 (0.998–2.306)	

**Fig. 4:**
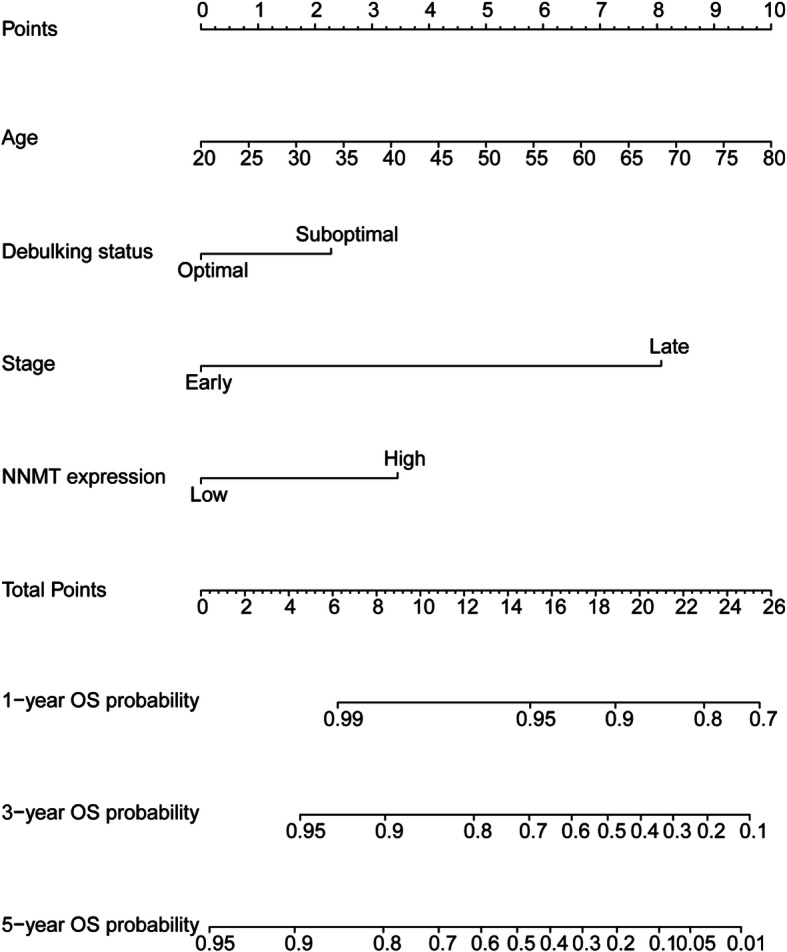
The NNMT-related nomogram developed from GSE9891 dataset. The nomogram illustrated that NNMT had a larger contribution to OS than debulking status

**Fig. 5:**
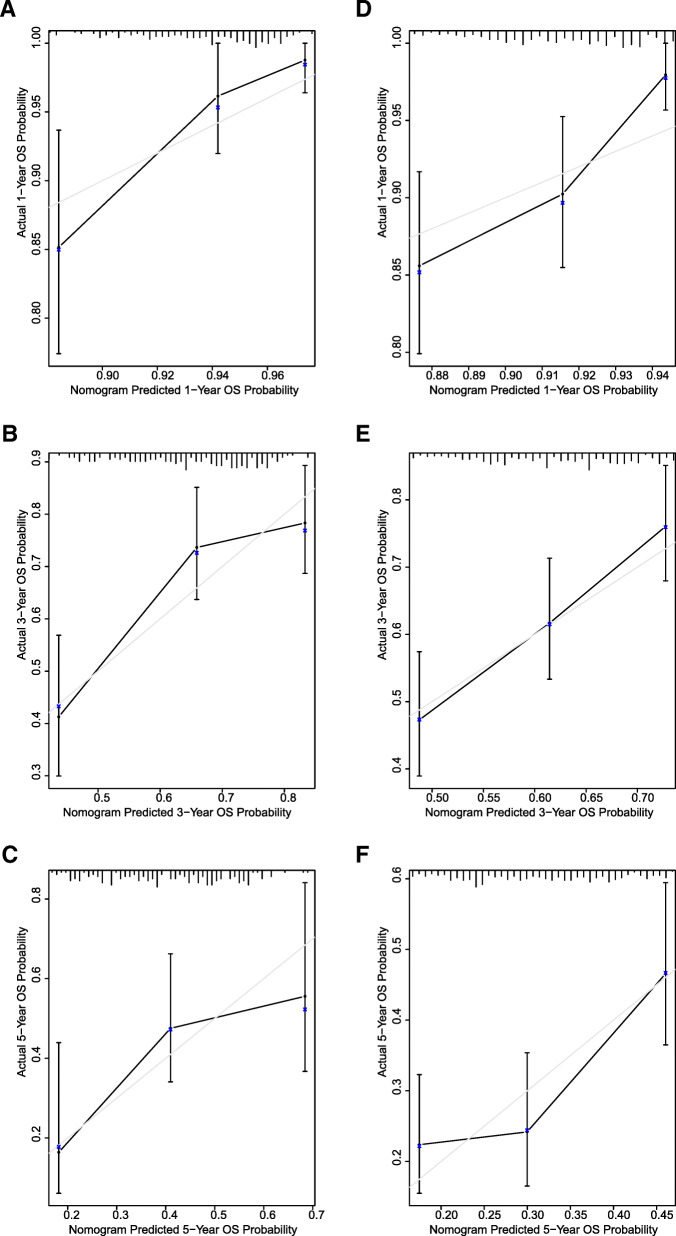
Calibration plots of the NNMT-related nomogram to predict OS. (A-C) Calibration plots of the NNMT-related nomogram to predict OS at 1 year (**a**), 3 years (**b**), and 5 years (**c**) in GSE9891 dataset. **d**-**f** Calibration plots of the NNMT-related nomogram to predict OS at 1 year (**d**), 3 years (**e**), and 5 years (**f**) in TCGA dataset

To quantitatively predict the survival of ovarian cancer patients, we developed a nomogram using GSE9891 dataset (training dataset) based on age at initial diagnosis, debulking status, FIGO stage, and NNMT expression, and validated it using TCGA dataset (validation dataset). Among the included factors, age at initial diagnosis had the largest contribution to OS, followed by FIGO stage and NNMT expression (Fig. 4). The C-index for predicting OS was 0.70 (95%CI, 0.64–0.75) in GSE9891 dataset (training dataset) and 0.64 (95%CI, 0.60–0.68) in TCGA dataset (validation dataset), respectively. The calibration plots demonstrated optimal agreement between the predicted and observed probability (Fig. 5a-f).

### The influence of NNMT expression on bevacizumab response

**Fig. 6:**
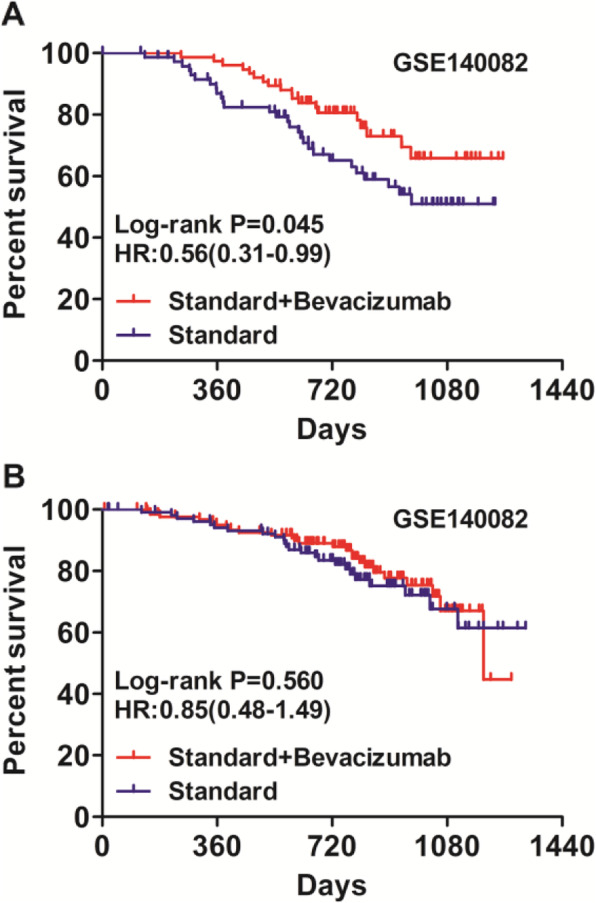
The association of NNMT expression with bevacizumab response in ovarian cancer patients. **a** Bevacizumab conferred significant improvements in OS for patients with low NNMT expression (HR: 0.56, 95%CI: 0.31–0.99, Log-rank *P* = 0.049). **b** Patients with high NNMT expression didn’t benefit from bevacizumab treatment significantly (HR: 0.85, 95%CI: 0.48–1.49, Log-rank *P* = 0.561)

Then, we explore whether NNMT expression was predictive of response to bevacizumab usding GSE140082 dataset. Interestingly, it was found that bevacizumab conferred significant improvements in OS for patients with low NNMT expression (Fig. 6a, HR: 0.56, 95%CI: 0.31–0.99, Log-rank *P* = 0.049). In contrast, patients with high NNMT expression didn’t benefit from bevacizumab treatment significantly (Fig. 6b, HR: 0.85, 95%CI: 0.48–1.49, Log-rank *P* = 0.561).

### The correlations of NNMT expression with the expression of genes involved in Warburg effect

**Fig. 7:**
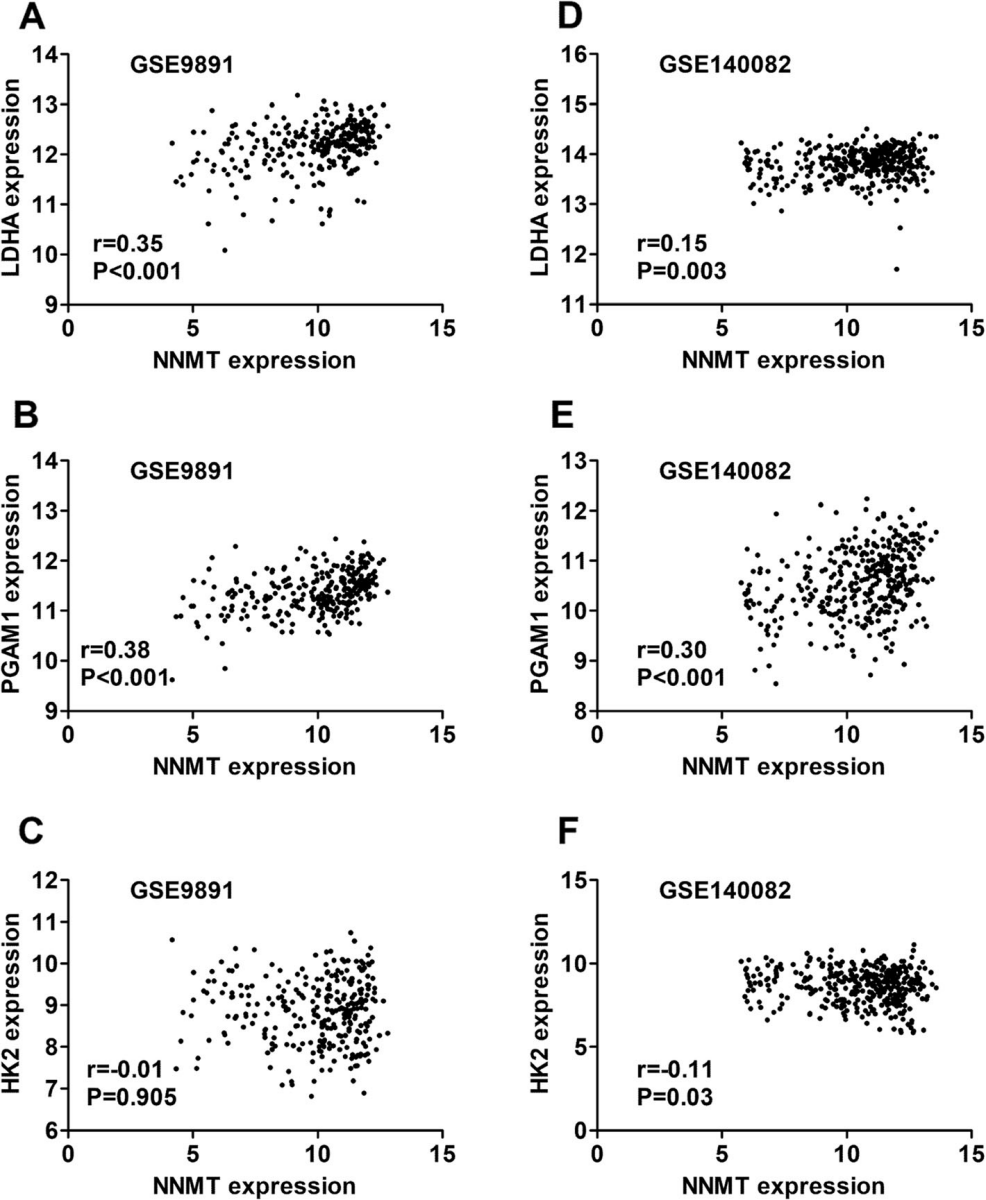
The correlations of NNMT expression with the expression of LDHA, PGAM1, and HK2. (A, B, C) The correlation of NNMT expression with LDHA expression (**a**), PGAM1 expression (**b**), and HK2 expression (**c**) in GSE9891 dataset. **d**, **e**, **f** The correlation of NNMT expression with LDHA expression (D), PGAM1 expression (**e**), and HK2 expression (**f**) in GSE140082 dataset

**Fig. 8:**
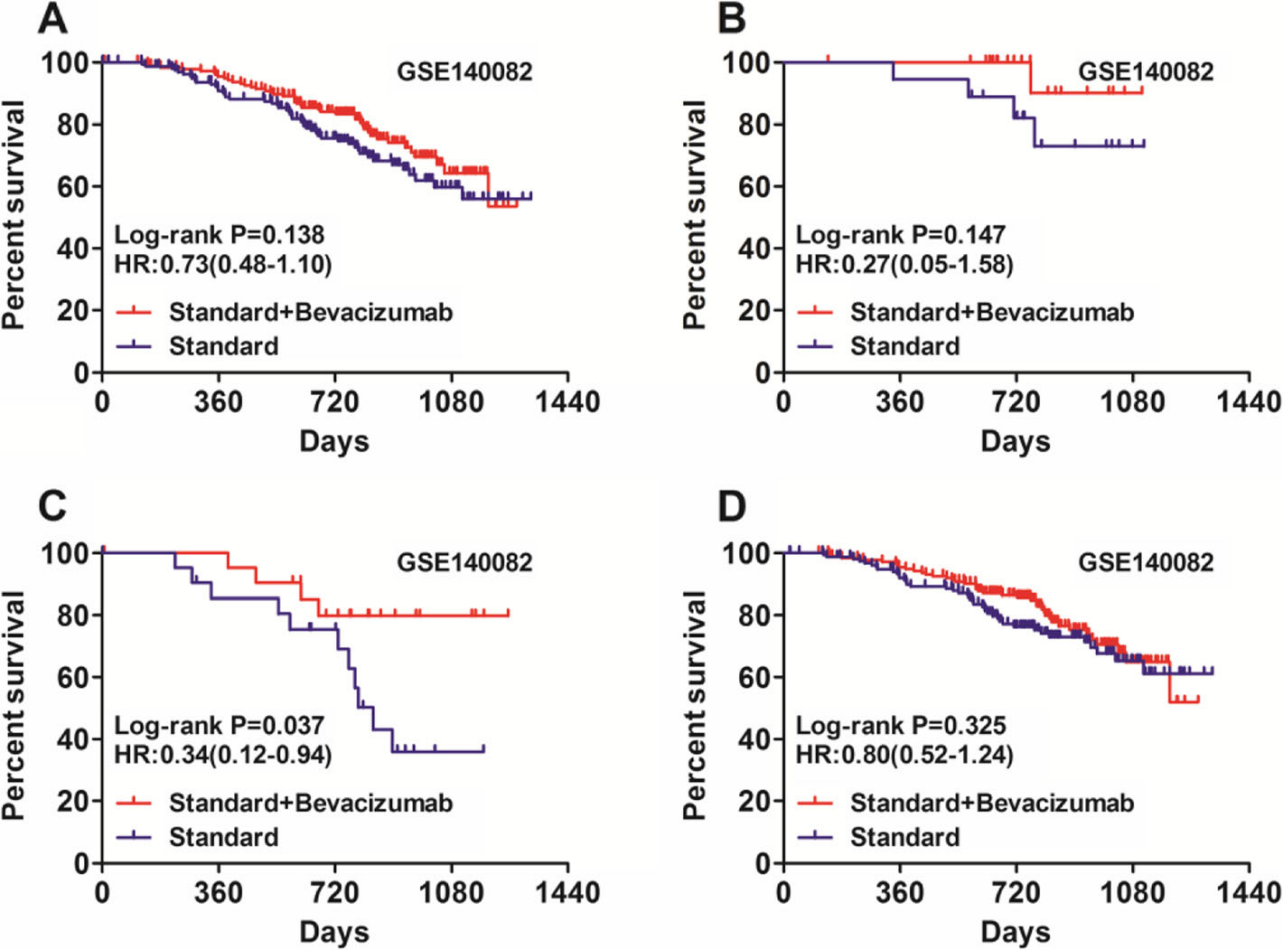
The associationS of LDHA and PGAM1 expression with bevacizumab response in ovarian cancer patients. **a**, **b** Bevacizumab conferred no significant improvements in OS for patients with both low LDHA expression (**a**) () and high LDHA expression (**b**) (). **c** Bevacizumab conferred significant improvements in OS for patients with low PGAM1 expression (HR: 0.34, 95%CI: 0.12–0.94, Log-rank *P* = 0.037). **d** Patients with high PGAM1 expression didn’t benefit from bevacizumab treatment significantly (HR: 0.80, 95%CI: 0.52–1.24, Log-rank *P* = 0.325)

Finally, we explore whether NNMT expression was correlated with the expression of genes (hexokinase 2 (HK2), LDHA, and PGAM1) involved in Warburg effect. Interestingly, our results suggested that NNMT expression was positively correlated with LDHA (Fig. 7a, r = 0.347, *P* < 0.001) and PGAM1 (Fig. 7b, r = 0.38, *P* < 0.001) expression, but not with HK2 expression (Fig. 7c, r = − 0.01, *P* = 0.905). The positive correlations of NNMT expression with the expression of LDHA (Fig. 7d, r = 0.15, *P* = 0.003) and PGAM1 (Fig. 7e, r = 0.30, *P* < 0.001) were confirmed in GSE140082 dataset. LDHA expression was not associated with bevacizumab response in ovarian cancer patients (Fig. 8a, Fig. 8b). However, bevacizumab conferred significant improvements in OS for patients with low PGAM1 expression (Fig. 8c, HR: 0.34, 95%CI: 0.12–0.94, Log-rank *P* = 0.037). In contrast, patients with high PGAM1 expression didn’t benefit from bevacizumab treatment significantly (Fig. 8d, HR: 0.80, 95%CI: 0.52–1.24, Log-rank *P* = 0.325).

## Discussion

The major findings of the present study can be summarized as follows. First, NNMT expression was significantly increased in LVSI-positive ovarian cancer tissues compared with LVSI-negative ovarian cancer tissues. Second, increased expression of NNMT was associated with increased tumor stage, grade, and mesenchymal molecular subtype. Third, higher NNMT expression correlated with unfavorable outcomes in ovarian cancer patients. Finally, NNMT expression was predictive of response to bevacizumab in ovarian cancer patients.

Recently, NNMT expression has been associated with a mesenchymal signature in bladder cancer and renal cancer [7, 8]. Consistently, our results also indicated that NNMT expression was increased in mesenchymal molecular subtype of ovarian cancer compared with other molecular subtypes of ovarian cancer. Functional studies revealed that knockdown of NNMT expression resulted in reduced migration and invasion of various cancer cells, suggesting an pro-metastatic role of NNMT [7, 8, 18, 19]. Consistent with these findings, we also found that the mean expression value of NNMT was increased in metastatic tumor tissues compared with primary counterparts, suggesting a potential pro-metastatic role of NNMT in ovarian cancer. But the differences were not statistically significant.

Previous studies have connected the increased expression of NNMT in cancer patients with chemoresistance [20]. The inhibition of NNMT expression in colorectal cancer HT-29 cells diminishes 5-FU resistance, while upregulation of NNMT in SW480 cells enhances it [20]. Our results also indicated that the mean expression value of NNMT was increased in drug resistant patients compared with drug sensitive patients, suggesting a potential link between NNMT elevation and drug resistance. But the differences were not statistically significant.

The prognostic value of NNMT has also been observed in various cancers. For example, increased expression of NNMT was associated with a worse prognosis in gastric cancer [19] and pancreatic cancer [21]. Our data also showed that NNMT abundance was associated with aggressive disease characteristics such as advanced stage and poor differentiation. More importantly, increased NNMT expression was linked to a reduced OS. Moreover, our NNMT-related nomogram showed that NNMT shared a larger contribution to OS, compared with debulking status. Previously, NNMT was identified as a novel serum marker colorectal cancer. Evaluation of the possibility of NNMT as serum marker for ovarian cancer is also needed.

Our previous work indicated that the presence of LVSI was associated with a worse clinical outcome in ovarian cancer [22]. LVSI is the first step of hematogenous metastasis of cancer cells [23], and we speculated that the expression of LVSI associated genes might be predictive of response to anti-angiogenesis therapy. Our work revealed that NNMT expression was higher in ovarian cancer tissues with LVSI than those without LVSI [24]. Thus, we explored whether the expression of LVSI associated gene, NNMT, was predictive of response to bevacizumab, an anti-angiogenesis agent which has been added to standard first-line chemotherapy in ovarian cancer [25, 26]. Interestingly, our results indicated that bevacizumab conferred significant improvements in OS for patients with low NNMT expression. In contrast, patients with high NNMT expression didn’t benefit from bevacizumab treatment significantly. Therefore, more survival benefit from bevacizumab treatment in ovarian cancer may be expected if it is used in patients with lower NNMT expression. And in patients with higher NNMT expression, combined therapy with NNMT inhibitor and bevacizumab may be translated into survival improvements.

Previous studies have indicated that NNMT is a master regulator in cancer cell metabolism in general and Warburg effect in particular [13, 14]. NNMT was an up-regulator of PGAM1, which was involved in Warburg effect [14]. Our data also demonstrated that NNMT expression was positively correlated with PGAM1 expression in ovarian cancer. Consistently, PGAM1 was also associated with bevacizumab response in ovarian cancer. This finding link NNMT expression to Warburg effect in particular. It may be helpful to clarify the mechanism of bevacizumab response in ovarian cancer.

## Conclusions

In conclusion, NNMT expression is associated with the aggressive behavior of ovarian cancer, correlates with a poor prognosis, and is predictive of sensitivity to bevacizumab treatment. Future researches are needed to establish the role of NNMT in ovarian cancer development, and to explore whether combined therapy with NNMT inhibitor and bevacizumab could be translated into survival improvements.

## Data Availability

The datasets analysed during the current study are available in GEO database and TCGA database.

## Abbreviations

NNMT: Nicotinamide N-methyltransferase
LDHA: Lactate dehydrogenase A
PGAM1: Phosphoglycerate mutase 1
HK2: Hexokinase 2
FTE: Fallopian tube epithelium
POCs: Primary ovarian cancers
MOCs: Metastatic ovarian cancers
ROCs: Recurrent ovarian cancers
LVSI: Lymphovascular space invasion
OS: Overall survival
